# Association between the Seroprevalence of Antibodies against Seasonal Alphacoronaviruses and SARS-CoV-2 Humoral Immune Response, COVID-19 Severity, and Influenza Vaccination

**DOI:** 10.3390/jcm12051733

**Published:** 2023-02-21

**Authors:** Lidia Brydak, Dominika Sikora, Barbara Poniedziałek, Ewelina Hallmann, Karol Szymański, Katarzyna Kondratiuk, Piotr Rzymski

**Affiliations:** 1 Department of Influenza Research, National Influenza Center at the National Institute of Public Health NIH—National Research Institute in Warsaw, 00-791 Warsaw, Poland; 2 Department of Environmental Medicine, Poznań University of Medical Sciences, 60-806 Poznan, Poland; 3 Doctoral School, Poznan University of Medical Sciences, 61-701 Poznan, Poland; 4 Integrated Science Association (ISA), Universal Scientific Education and Research Network (USERN), 60-806 Poznan, Poland

**Keywords:** seasonal coronaviruses, heterologous protection, pandemic

## Abstract

The present study assesses the seroprevalence of antibodies against seasonal human alphacoronaviruses 229E and NL63 among adult patients infected with SARS-CoV-2, and its association with the humoral response to SARS-CoV-2 infection and its severity, and influenza vaccination. A serosurvey was conducted to quantify the presence of IgG antibodies against the nucleocapsid of 229E (anti-229E-N) and NL63 (anti-NL63-N), and anti-SARS-CoV-2 IgG antibodies (against nucleocapsid, receptor-binding domain, S2 domain, envelope, and papain-like protease) for 1313 Polish patients. The seroprevalence of anti-229E-N and anti-NL63 in the studied cohort was 3.3% and 2.4%. Seropositive individuals had a higher prevalence of anti-SARS-CoV-2 IgG antibodies, higher titers of the selected anti-SARS-CoV2 antibodies, and higher odds of an asymptomatic SARS-CoV-2 infection (OR = 2.5 for 229E and OR = 2.7 for NL63). Lastly, the individuals vaccinated against influenza in the 2019/2020 epidemic season had lower odds of seropositivity to 229E (OR = 0.38). The 229E and NL63 seroprevalence was below the expected pre-pandemic levels (up to 10%), likely due to social distancing, increased hygiene, and face masking. The study also suggests that exposure to seasonal alphacoronaviruses may improve humoral responses to SARS-CoV-2 while decreasing the clinical significance of its infection. It also adds to accumulating evidence of the favorable indirect effects of influenza vaccination. However, the findings of the present study are of a correlative nature and thereby do not necessarily imply causation.

## 1. Introduction

Common seasonal human coronaviruses (sHCoVs) include two types belonging to the *Alphacoronavirus* genus (229E and NL63) and two representatives of *Betacoronavirus* (HKU1 and OC43). Contrary to the highly pathogenic severe acute respiratory syndrome coronavirus (SARS-CoV), Middle East respiratory syndrome-related coronavirus (MERS-CoV), and SARS-CoV-2 (all the belonging to the betacoronaviruses), their infections are predominantly associated with the upper respiratory tract and significantly lower severity, with the most frequently reported symptoms including rhinorrhea, nasal congestion, sore throat, headache, sneezing, fatigue, and cough that may be associated with a fever [1,2,3,4,5]. Therefore, the vast majority of cases do not require any specific treatment. Rarely, an infection of the lower respiratory tract can also occur, particularly in infants, immunocompromised patients, or the elderly [6,7,8,9,10]. Very rarely, acute gastroenteritis and neuroinvasion were also reported as a consequence of sHCoV infection [11,12].

In the temperate zone, the circulation of these viruses reveals a seasonal pattern with an increased frequency of infections in the autumn–winter period [13,14,15]. In general, 5–10% of upper respiratory tract infections in adults are caused by sHCoVs [16], while in children, they are responsible for 4–6% of all acute respiratory infections requiring hospitalization [17,18,19], and 8% infections in an ambulatory setting [18,20,21]. After rhinoviruses, the sHCoVs may be the second most frequent cause of the common cold [22,23].

sHCoVs are not subject to routine diagnostic testing, since their infections, contrary to SARS-CoV-2, do not represent a threat of acute respiratory distress syndrome, cytokine storm, thrombosis, and organ failure, and do not cause long-term consequences. Therefore, there are no accurate epidemiological data on the frequency of their infections in many regions, including Poland. During the COVID-19 pandemic, sHCoVs received increased attention due to reports suggesting the potential cross-reactivity of antibodies against sHCoVs and SARS-CoV-2 [24,25]. An in silico investigation indicated that OC43, HKU1, 229E, and NL63 may induce immune memory against SARS-CoV-2 via antigen epitopes for presentation in the context of MHC class I [26]. Therefore, previous infections with sHCoVs could elicit cross-protection against SARS-CoV-2. However, conducted studies yielded inconclusive and inconsistent results [27,28,29,30]. Such protective cross-reactivity may be more plausible in the case of sHCoVs belonging to betacoronaviruses (HKU1 and OC43) than to alphacoronaviruses (229E and NL63) due to the greater genetic similarity to SARS-CoV-2. In line with this, selected analyses demonstrated that high levels of antibodies against the OC43 nucleocapsid decrease the odds of becoming SARS-CoV-2-seropositive and were associated with lower disease severity [31,32]. Conversely, some studies showed that previous humoral immunity to 229E and NL63 correlates with worse clinical outcome in COVID-19, suggesting that they may be responsible for antibody-dependent enhancement during SARS-CoV-2 infection [33,34].

At the same time, the decreased social mobility and sanitary measures could have affected the circulation of sHCoVs during the COVID-19 pandemic similarly to various other respiratory viruses, such as influenza A and B viruses, respiratory syncytial virus, metapneumovirus, adenoviruses, and parainfluenza viruses [35,36,37,38]. Therefore, the need remains to explore the association between the exposure to sHCoVs and SARS-CoV-2, and the factors that may influence this potential relationship. Considering that the vast majority of infections with sHCoVs are not confirmed using diagnostic tests (e.g., real-time reverse transcription polymerase chain reaction test), their circulation can be assessed with surveys based on antibody-based serology. Despite this, for various regions such as Central and Eastern Europe, the data on the seroprevalence of sHCoVs before and during the COVID-19 pandemic are scarce and require updating.

The present study assesses the seroprevalence of IgG antibodies against 229E and NL63 in the Polish population of adults who had undergone SARS-CoV-2 infection during the COVID-19 pandemic, and analyzes whether the presence of antibodies against sHCoVs is associated with COVID-19 severity and the humoral response to SARS-CoV-2 infection. Moreover, since the vaccination against influenza had been associated with lower odds of infection with SARS-CoV-2 [39,40], we aimed to understand whether such a relationship may also exist in the case of seasonal human alphacoronaviruses.

## 2. Material and Methods

### 2.1. Sample Collection

The analysis included serum samples from 1313 patients that had been purchased from regional blood donation and blood treatment centers located across Poland in eight cities (Białystok, Warsaw, Radom, Racibórz, Kalisz, Bydgoszcz, Łódź, Szczecin, and Wrocław). All samples were collected between September and December 2020 from SARS-CoV-2-infected patients (confirmed with RT-PCR) 1 month (±2 weeks) after the resolution of the symptoms/end of the isolation period. According to the Global Initiative on Sharing All Influenza Data, during March and December 2020, SARS-CoV-2 infections in Poland were primarily caused by Nextstrain clades 20A, 20B, 20D, and 20G that did not reveal major differences in clinical outcomes [41,42,43]. Until September 2020, the numbers of confirmed SARS-CoV-2 infections and COVID-19 deaths in Poland reached 95,772 and over 1.3 million, respectively, and increased to 2569 and 28,955, respectively, by the end of December 2020 [44]. In response to the pandemic, a strict nationwide lockdown was imposed by the Polish government in March 2020, with the relaxation of some measures introduced at the beginning of May 2020. However, in the first half of April 2020, mandatory face mask use in public spaces was introduced. On 17 October 2020, novel social distancing measures were imposed, dividing a country into two types of zones: “yellow” (mandatory face masking in public spaces, capacity restrictions applied for public transportation, restaurants, cultural events, public gatherings, and religious areas, hybrid learning at universities and secondary schools, sports events held without an audience) and “red” (compared to “yellow” zones, tighter capacity restrictions for public transportation, commercial facilities, public assemblies, and religious services; universities and schools were required to hold classes online.). However, from 23 October, the lockdown measures were tightened again, with kindergartens and school closures, and social mobility was reduced only to essential needs. Therefore, between March and December 2020, the circulation of infectious respiratory agents, including sHCoVs, was significantly altered.

The data on age, sex, COVID-19 severity (divided into three groups: (i) asymptomatic infection, (ii) mild infection not requiring hospitalization, and (iii) severe infection requiring hospitalization), and influenza vaccination status in the 2019/2020 epidemic season were available for each included patient (Table 1). 

All influenza-vaccinated individuals received the vaccine in the recommended period between September and December 2019, approximately one year prior to infection with SARS-CoV-2. During the 2019–2020 epidemic season, an inactivated quadrivalent influenza vaccine contained the following antigens: (i) an A/Brisbane/02/2018 (H1N1)pdm09-like virus, (ii) an A/Kansas/14/2017 (H3N2)-like virus, (iii) a B/Colorado/06/2017-like virus (B/Victoria/2/87 lineage), and (iv) a B/Phuket/3073/2013-like virus) [45]. None of the patients included in the present study had been vaccinated against COVID-19 (the national vaccination campaign in Poland began in January 2021) [46].The research was approved by the Bioethical Committee of the Institute of Public Health, National Research Institute (approval no. 4/2020; date of approval: 6 August 2020) and the Bioethics Committee at Poznan University of Medical Sciences (approval no. 429/22; date of approval: 11 May 2022). The study was performed in accordance with the ethical standards as laid down in the 1964 Declaration of Helsinki and its later amendments. Patient consent was waived because serum samples for research were purchased from Polish regional blood donor centers according to the accepted safeguard standards and legal requirements in Poland.

### 2.2. Determination of Antibodies

IgG antibodies against the nucleocapsid protein of 229E (anti-229E-N) and NL63 (anti-NL63-N) in serum samples were quantified with CE-IVD certified (indicating that it complies with the European In-Vitro Diagnostic Devices Directive, IVDD 98/79/EC) Microblot-Array COVID-19 IgG assay (TestLine Clinical Diagnostics, Brno, Czech Republic). The same test was used to determine anti-SARS-CoV-2 IgG antibodies against the nucleocapsid protein (anti-N), a receptor-binding domain of the spike protein (anti-RBD), subunit S2 of the spike protein (anti-S2), an envelope protein (anti-E), and papain-like protease protein (anti-PLpro). The same test also measures autoantibodies against angiotensin-converting enzyme 2, but these were reported elsewhere for lower sample size [47]. In this assay, recombinant and purified native antigens are immobilized on specific spots of nitrocellulose membrane fixed at the bottom of the microplate well. According to the information provided by the manufacturer, the assay demonstrates diagnostic sensitivity of 98.7% and diagnostic specificity of 99.3%. In all determinations, the concentration of antibodies is reported as U/mL and considered positive if above 210 U/mL according to the manufacturer’s instructions.

### 2.3. Statistical Analysis

Statistical analysis was conducted using Statistica v.13.3 (StatSoft Inc., Tulsa, OK, USA). Comparing the patients’ characteristics (sex, COVID-19 severity, status of influenza vaccination in the 2019/2020 epidemic season, frequency of SARS-CoV-2 seroconversion) and prevalence of anti-SARS-CoV-2 antibodies between 2293E/NL63 seropositive and seronegative groups was assessed with Pearson’s chi-squared (χ^2^) test. The Mann-Whitney U test was used to evaluate the difference in age between these groups and to test the differences in anti-229E-N and anti-NL63-N concentrations in relation to age (>50 and ≤50 years), sex, influenza vaccination status, and severity of SARS-CoV-2 infection among patients seropositive for seasonal alphacoronaviruses. In addition, to assess the relationship between antibody prevalence and the clinical course of SARS-CoV-2 infection, and the status of influenza vaccination, the classical odds ratio with a 95% confidence interval (95%CI) was calculated using MedCalc (MedCalc, Ostend, Belgium). The differences were considered statistically significant at a *p*-value < 0.05.

## 3. Results

### 3.1. Seroprevalence of IgG Antibodies against Seasonal Alphacoronaviruses

The overall prevalence of IgG anti-229E-N antibodies was 3.3%, with mean + SD titers of 332.7 ± 171.0 U/mL. In the case of IgG anti-NL63-N antibodies, the prevalence and concentration were 2.4% and 329.0 ± 136.6 U/mL, respectively.

### 3.2. Association between Antibodies against Seasonal Alphacoronaviruses and Humoral Response to SARS-CoV-2 Infection

**Figure 1:**
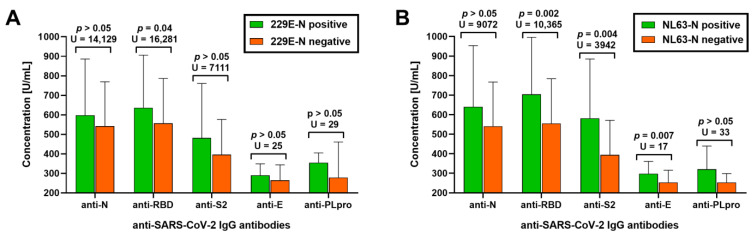
Serum titers of IgG antibodies against SARS-CoV-2 nucleocapsid (anti-N), a receptor-binding domain of spike protein (anti-RBD), subunit S2 of spike protein (anti-S2), an envelope protein (anti-E), and papain-like protease (anti-PLpro) in individuals seropositive and seronegative to (**A**) alphacoronavirus 229E and (**B**) NL63.

The subjects with detectable IgG anti-229E-N and IgG anti-NL43-N had a significantly higher prevalence of all anti-SARS-CoV-2 immunoglobulins, including a 12-fold higher frequency of IgG anti-E and IgG anti-PLpro IgG antibodies (Table 2). Nearly all (93%) individuals with IgG anti-229E-N antibodies and all (100%) with IgG anti-NL63-N antibodies had undergone SARS-CoV-2 seroconversion. Exposure to at least one of the seasonal alphacoronaviruses was associated with higher odds for SARS-CoV-2 seroconversion (OR = 3.7, 95%CI: 1.2–11.9, *p* = 0.03). Moreover, being seropositive to sHCoV was associated with increased titers of selected IgG anti-SARS-CoV-2 antibodies among seroconverted individuals (Figure 1). The seropositive group to 299E had higher concentrations of IgG anti-RBD antibodies (by 14%), while NL63-seropositive subjects revealed higher levels of IgG anti-RBD (by 26%), IgG anti-S2 (by 48%), and IgG anti-E (by 17%) antibodies.

### 3.3. Factors Associated with the Prevalence of Antibodies against Seasonal Alphacoronaviruses

Age and sex did not affect the prevalence of tested antibodies (Table 3). A higher prevalence of asymptomatic SARS-CoV-2 infections was noted among those who had tested positive for IgG anti-229E-N or IgG anti-NL63-N (Table 3).

The odds of being asymptomatic in these groups were increased (OR = 2.5, 95%CI: 1.3–4.5; *p* = 0.004 and OR = 2.7, 95%CI: 1.3–5.5; *p* = 0.008, respectively). The titers of IgG anti-229E-N and IgG anti-NL63-N antibodies did not differ between groups with = different clinical courses of SARS-CoV-2 infection (asymptomatic, mild and not hospitalized, severe and hospitalized). Patients vaccinated against influenza in the 2019/2020 epidemic season had a lower prevalence of IgG anti-229E-N antibodies (OR = 0.38, 95%CI: 0.19–0.74; *p* = 0.005) but not IgG anti-NL63-N (OR = 1.1, 95%CI: 0.52–2.2; *p* > 0.05) (Table 2). Age, sex, COVID-19 severity, and influenza vaccination status did not differentiate the concentrations of IgG anti-229E-N and IgG anti-NL63-N antibodies (*p* > 0.05 in all cases).

## 4. Discussion

This study indicates that the seroprevalence of antibodies against common seasonal alphacoronaviruses 229E and NL63 in Polish adults was low during the COVID-19 pandemic. Although there are no reference data on the national level to which the findings of our study could be compared, the epidemiological analysis conducted in France indicated that, during the pre-pandemic influenza surveillance seasons, sHCoV infections affected up to 10% of the population, with the highest share of OC43 cases followed by NL63, HKU1, and eventually 229E [48]. In general, most adults have been exposed to at least one sHCoV, but humoral immunity is short-lived, with reinfections most frequently observed after 12 months post-infection. For example, one US study reported that the number of sHCoV infections experienced over the 2010–2018 period ranged from 1 to 13 per individual, with some patients facing reinfection during the same study year [49]. In other words, the seroprevalence observed in our study was likely lower than that expected before COVID-19. This most plausibly resulted from the diminished circulation of various infectious agents due to various sanitary measures (e.g., hand-washing, face masking) and social distancing. Previous analysis indicated that, during the COVID-19 pandemic, the incidence of various infectious diseases in Poland significantly decreased compared to the 2015–2019 period [50]. According to one analysis conducted among hospitalized patients in Italy, compared to the pre-pandemic period, the prevalence of sHCoV infections during the pandemic decreased over 35-fold [37].

Notably, patients positive for IgG antibodies against 229E-N and NL63-N revealed a significantly higher prevalence of all anti-SARS-CoV-2 IgG antibodies following the infection, including rarely detected anti-E and anti-PLpro immunoglobulins. This finding is in line with a previous observation that pre-existing IgG antibodies against the sHCoV spike protein are correlated with higher IgG antibody titers against SARS-CoV-2, with generally more profound association found for betacoronaviruses OC43 and HKU1; this was also demonstrated for 229E and NL63 [27]. Such an association was also observed in the case of SARS-CoV [51].

Such a finding may have two implications. First, a better humoral response may translate into higher protection against reinfection or exert improved neutralization effects if reinfection occurs [52]. On the other hand, the magnitude of antibody responses to SARS-CoV-2 infection was an indicator of severity, with lower seroconversion in asymptomatic cases and the greatest humoral responses in severe cases [53,54]. Considering that some studies demonstrated that pre-existing antibodies to 229E and NL63 may be associated with worse clinical outcome in COVID-19, they may be responsible for the antibody-dependent enhancement during SARS-CoV-2 infection [33], ultimately resulting in improved humoral response against SARS-CoV-2 in survivors. Contrary to these observations, the present study found that seropositivity to both 229E and NL63 was associated with higher odds of asymptomatic SARS-CoV-2 infection, indicating the potential cross-protective effect of exposure to seasonal alphacoronaviruses. Notably, we employed samples collected from convalescent patients with a wider variety of clinical courses of SARS-CoV-2 infection, from asymptomatic to severe, requiring hospitalization, whereas the above-referenced studies focused on a group of symptomatic patients admitted to the hospital. Therefore, our results may better reflect the generalized association between the exposure to seasonal alphacoronaviruses and the severity of SARS-CoV-2 infection.

Moreover, our study found an association between vaccination against influenza in the 2019/2020 epidemic season and the lower seroprevalence of IgG antibodies against 229E-N. Previous epidemiological analyses indicated some potential cross-protective effects of influenza vaccination against SARS-CoV-2, including lower odds of SARS-CoV-2 infection, hospitalization, the need for mechanical ventilation, and death due to COVID-19, and revealed improved humoral responses [39,40,55,56]. Serological analyses also emphasize the role of anti-hemagglutinin antibodies in the course of SARS-CoV-2 infection [57]. This phenomenon is likely a result of the vaccination-induced training of innate immunity, a process of the epigenetic reprogramming of transcriptional pathways that ultimately allows for the innate immune system to exhibit adaptive characteristics [58,59]. A similar cross-protective effect may also occur in the context of sHCoVs, as suggested by the present study’s findings, adding to the accumulating evidence on the broadly beneficial effects of influenza vaccination against heterologous infections [40,56,60]. Such benefits that go beyond protection from influenza are an addition that could convince the general public to be vaccinated against influenza, particularly in regions characterized by poor uptake, such as Poland, where the coverage rate is usually <5% in the general population and also remains low in the group of healthcare workers [61,62,63].

### 4.1. Study Strengths

Our study has several strengths that support the validity of our findings. It included a relatively large group of adult patients and reports on the seroprevalence of antibodies against seasonal alphacoronaviruses for the Polish population, for which such data had not been available so far. In fact, the availability of such data for neighboring countries is also limited. Therefore, the present results may serve as a reference point for future research on the circulation of these pathogens in Central Europe and the humoral responses to their infections. Furthermore, apart from the alphacoronaviral context, our study reported on the prevalence of rarely investigated anti-SARS-CoV-2 antibodies, namely, IgG anti-E and IgG anti-PLpro. Moreover, contrary to other studies, our research was conducted on serum samples collected within a specific time frame of the COVID-19 pandemic: during the autumn–winter season of 2020, after the national lockdown, and during a period when strict sanitary measures and social distancing had been imposed and practiced. Therefore, the observed seroprevalence, below the expected levels, indicates that the dynamics of seasonal respiratory viruses, such as 229E and NL63, were significantly altered. This effect may be viewed as beneficial, although it may also indicate that, with lifted restrictions, the resurgence of infections caused by these viruses can be expected in the future. Various investigations conducted in 2021 and autumn–winter 2022 already evidenced that the relaxation of COVID-19 containment measures was associated with an unusual increase in respiratory infections caused by agents other than SARS-CoV-2, e.g., respiratory syncytial virus or human metapneumovirus [64,65,66].

To the best of our knowledge, this is the first study to investigate the potential association between influenza vaccination and the prevalence of antibodies against seasonal alphacoronaviruses. Its findings advocate for future research exploring whether influenza vaccination may decrease the exposure to other pathogens through the potential cross-protective effects of trained innate immunity. Such studies should predominantly focus on viruses of which the increased circulation overlaps with influenza and vaccination against it, e.g., metapneumovirus, parainfluenza virus, respiratory syncytial virus, and rhinoviruses [15,67,68]. This could be approached by comparing the frequency of infections or antibody seroprevalence in vaccinated and unvaccinated groups against influenza, through experimental in vivo models (e.g., ferrets or rodents), or via in vitro studies employing immune cells collected from vaccinated individuals.

### 4.2. Study Limitations

We also wish to stress some limitations of our study. First, the seroprevalence of seasonal alphacoronaviruses was assessed in Polish patients who had contracted SARS-CoV-2, and although the susceptibility to infection with this pathogen was relatively similar among adults of different ages [69], our findings may not fully represent seroprevalence on the national level. Second, our study was not longitudinal or included the SARS-CoV-2-naive comparator group. Therefore, the findings are of a correlative nature, and as long as they may appear suggestive, they should not be used to imply causation. Third, due to the unavailability of data, this study did not include some patient characteristics that may also influence humoral responses to SARS-CoV-2, such as body mass index, specific comorbidities, or the use of medications. Moreover, our study did not investigate the function of anti-SARS-CoV-2 antibodies. Therefore, whether the higher antibody concentrations found for 299E and NL63 seropositive individuals would be associated with better viral neutralization requires exploration through further research. However, increased titers of anti-RBD IgG antibodies, which were higher in groups exposed to seasonal alphacoronaviruses, are associated with a significantly reduced risk of reinfection [70]. Although individuals exposed to 229E and NL63 being more likely to undergo asymptomatic SARS-CoV-2 infection potentially indicates the involvement of cross-protective adaptive cellular immunity, this was not subject to our study and would require further investigations. In addition, the study was conducted before the emergence of SARS-CoV-2 variants of concern, such as Alpha (B.1.1.7 lineage), Delta (B.1.617.2 lineage), and Omicron (B.1.1.529 lineage), which revealed genomic, biological, and clinical differences compared to viral variants circulating in 2020 [41,71,72]. Whether these differences could have an effect on the associations evidenced in our research requires further investigation, which we highly encourage.

Lastly, our findings are solely related to the association between the exposure to seasonal alphacoronaviruses and SARS-CoV-2 infection. It remains to be understood whether COVID-19 vaccination in subjects exposed to sHCoVs could also translate into improved humoral responses.

## 5. Conclusions

The seroprevalence of HCoV-229E and HCoV-NL63 in the studied cohort was low, likely due to the social-distancing and sanitary measures during the COVID-19 pandemic. Individuals who had tested positive for seasonal alphacoronaviruses had a better humoral response to SARS-CoV-2 infection and were more likely to be asymptomatic, indicating potential cross-protective effects. Influenza vaccination was related to a decreased risk of being seropositive to NL63 alphacoronavirus, again suggesting the potential cross-protective effects of influenza vaccination against heterologous infection. However, the findings of the present study are of a correlative nature; therefore, they do not necessarily imply causation.

## Figures and Tables

**Table 1:** Demographic characteristics of the studied group of patients (*n* = 1313) whose serum samples were used in the analysis.

Age, mean ± SD (min–max)	37.2 ± 9.8 (18–80)
>50 years, % (*n*)	9.8 (129)
Women/men, % (*n*)	20.3 (266)/79.7 (1047)
COVID-19 severity	
- Asymptomatic, % (*n*)	27.6 (363)
- Mild, not hospitalized, % (*n*)	66.2 (869)
- Severe, hospitalized, % (*n*)	6.2 (81)
Influenza vaccination in the 2019/2020 season	
- Vaccinated, % (*n*)	50.1 (658)
- Not vaccinated, % (*n*)	49.9 (655)

**Table 2:** Prevalence of anti-SARS IgG antibodies in patients testing seropositive and seronegative for IgG antibodies against 229E and NL63 nucleocapsid (N).

	IgG anti-229E-N		IgG anti-NL63-N	
	Positive (*n* = 43)	Negative (*n* = 1270)	Positive (*n* = 31)	Negative (*n* = 1282)
**anti-N**	86.0	67.3	83.9	67.6
	*p* = 0.01 (χ^2^ = 6.7)		*p* = 0.05 (χ^2^ = 3.7)	
**anti-RBD**	93.0	77.9	100.0	77.8
	*p* = 0.02 (χ^2^ = 5.6)		*p* = 0.003 (χ^2^ = 8.8)	
**anti-S2**	72.1	41.3	74.2	41.5
	*p* < 0.001 (χ^2^ = 16.2)		*p* < 0.001 (χ^2^ = 13.3)	
**anti-E**	34.9	0.55	45.2	0.62
	*p* < 0.001 (χ^2^ = 297.6)		*p* < 0.001 (χ^2^ = 364.4)	
**anti-PLpro**	37.2	0.47	48.4	0.54
	*p* < 0.001 (χ^2^ = 340.2)		*p* < 0.001 (χ^2^ = 420.5)	

**Table 3:** Main characteristics of patients with IgG antibodies against 229E and NL63 nucleocapsid (N).

	IgG anti-229E-N		IgG anti-NL63-N	
	Positive (*n* = 43)	Negative (*n* = 1270)	Positive (*n* = 31)	Negative (*n* = 1282)
Age (mean ± SD)	37.6 ± 9.5	37.2 ± 9.8	35.8 ± 9.9	37.2 ± 9.8
	*p* > 0.05 (*U* = 26,450)		*p* > 0.05 (*U* = 18,022)	
Women/men (%)	20.9/79.1	20.3/79.7	25.8/74.2	20.2/79.8
	*p* > 0.05 (χ^2^ = 0.01)		*p* > 0.05 (χ^2^ = 0.6)	
Vaccinated against influenza/unvaccinated (%)	27.9/72.1	50.9/49.1	51.6/48.4	50.1/49.9
	*p* = 0.003 (χ^2^ = 8.8)		*p* > 0.05 (χ^2^ = 0.03)	
COVID-19 severity - Asymptomatic (%) - Mild (%) - Severe (%)				
	48.8	26.9	48.4	27.1
	48.8	66.8	48.4	66.6
	2.3	6.3	3.2	6.2
	*p* = 0.006 (χ^2^ = 10.3)		*p* = 6.9 (χ^2^ = 6.9)	

## Data Availability

Data supporting reported results can be provided upon request from the corresponding author.
